# AAVR Expression is Essential for AAV Vector Transduction in Sensory Hair Cells

**DOI:** 10.1002/advs.202408873

**Published:** 2025-01-07

**Authors:** Fan Wu, Guisheng Chen, Rui Hu, Peiwen Liu, Jintao Lou, Wenji Zhao, Zuhong He, Suhua Sha, Yiqing Zheng

**Affiliations:** ^1^ Department of Otolaryngology Sun Yat‐sen Memorial Hospital Sun Yat‐sen University Guangzhou Guangdong 510120 China; ^2^ Department of Pathology and Laboratory Medicine The Medical University of South Carolina Walton Research Building, Room 403‐E, 39 Sabin Street Charleston SC 29425 USA; ^3^ Guangdong Provincial Key Laboratory of Malignant Tumor Epigenetics and Gene Regulation Sun Yat‐sen Memorial Hospital Sun Yat‐sen University Guangzhou Guangdong 510120 China; ^4^ Institute of Hearing and Speech‐Language Science Sun Yat‐sen University Guangzhou 510120 China; ^5^ Guangdong Provincial Key Laboratory of Cancer Pathogenesis and Precision Diagnosis and Treatment, Shenshan Medical Center Sun Yat‐sen Memorial Hospital, Sun Yat‐sen University Shanwei Guangdong 516621 China; ^6^ Department of Otorhinolaryngology‐Head and Neck Surgery Zhongnan Hospital of Wuhan University Wuhan 430071 China

**Keywords:** AAV receptor (Kiaa0319l, Au040320), AAV vector transduction in inner ear hair cells, AAVR knockout mice, AAVR conditional knock‐in in inner ear hair cells

## Abstract

Adeno‐associated virus (AAV) vectors are a leading platform for gene therapy. Recently, AAV‐mediated gene therapy in the inner ear has progressed from laboratory use to clinical trials, but the lower transduction rates in outer hair cells (OHCs) in the organ of Corti and in vestibular hair cells in adult mice still pose a challenge. OHCs are particularly vulnerable to inner ear insults. In this study, we demonstrated that expression of a key AAV receptor (AAVR, *Kiaa0319l*, *or Au040320*) in OHCs and vestibular hair cells decreases significantly in mature mice and AAV particles directly interact with AAVR by forming complexes. Consequently, antibody blockage of AAVR significantly inhibits AAV transduction in sensory hair cells in cochlear explants. Moreover, use of AAVR knockout mice confirms inhibition of AAV transduction in sensory hair cells in vivo. Finally, conditional overexpression of AAVR in sensory hair cells of adult mice successfully restores AAV transduction efficiency in OHCs and vestibular hair cells. In conclusion, this strong evidence that AAVR is essential for AAV transduction in sensory hair cells will help to increase the efficacy of future gene therapy in inner ear.

## Graphical abstract

Decreased sensitivity to AAV vector transduction in the outer hair cells (OHCs) of adult mice is primarily attributed to reduction of AAVR (Kiaa0319l; Au040320). Knockout of AAVR reduces AAV vector transduction efficiency in both inner hair cells (IHCs) and OHCs in neonatal mice. Conditional overexpression of AAVR in sensory hair cells restores the sensitivity to AAV transduction in adult mice.

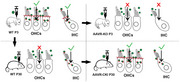

## 1 Introduction

Use of adeno‐associated virus (AAV) vectors for gene therapy has advanced rapidly over the past decade. Recent outcomes from clinical trials for treatment of patients with autosomal recessive deafness 9 (DFNB9) via AAV‐mediated gene therapy has gained significant attention worldwide.^[^ ^1^, ^2^, ^3^ ^]^ Although long‐term clinical data are still needed, the clinical hearing recovery achieved through AAV‐mediated gene therapy provides direct evidence that AAV vectors are a powerful platform for treatment of hearing loss in humans.

To date, while AAV‐based gene therapy has emerged as an important platform for treatment of genetic hearing loss, evidence is limited for its use against acquired hearing loss pathologies, such as age‐related, noise‐, and ototoxic drug‐induced hearing loss, possibly due to the limitation of AAV transduction in outer hair cells (OHCs) in adult age. Environmental factors contribute to acquired‐hearing loss in an unpredictable fashion over a lifespan.^[^ ^4^, ^5^, ^6^ ^]^ The main pathological feature of acquired hearing loss is loss of OHCs.^[^ ^7^, ^8^, ^9^ ^]^ As mature cochlear OHCs are unable to regenerate, loss of hair cells leads to permanent hearing loss. The suffering caused and the financial costs of acquired‐hearing loss highlight its treatment as a high‐priority health concern.^[^ ^10^ ^]^ Currently, no approved pharmaceutical therapy is available, due in part to the complexity of the cell death pathways induced by such noxious challenges and the uncertainty of potential drug targets^[^ ^9^, ^11^ ^]^ and utilization of AAV vectors may provide a novel therapeutic tool for treatment. Additionally, some genetic hearing loss syndromes are associated with gene mutations in OHCs,^[^ ^12^, ^13^ ^]^ therefore, enhancing AAV infection in OHCs could widen the treatment window for both acquired and genetic hearing loss.^[^ ^14^, ^15^ ^]^

Enhancing AAV transduction rates in target cells is essential for improved functional recovery. Reconstruction of capsids with novel serotypes, such as AAV1, AAV2, and AAV9, along with engineered capsids like Anc80L65, AAV2.7m8, AAV‐ie, and AAV‐PHP.eB, has increased AAV transduction efficiency in targeted inner ear cells of neonatal mice.^[^ ^16^, ^17^, ^18^, ^19^ ^]^ However, challenges persist in increasing infection efficiency in OHCs at adult ages.^[^ ^17^, ^20^, ^21^, ^22^ ^]^ Although some AAV serotypes have been used to achieve partial transduction in OHCs in adult mice through increased viral concentration or enhanced injection techniques,^[^ ^20^, ^23^, ^24^, ^25^ ^]^ most studies have found that AAV transduction efficiency in cochlear OHCs is far lower than that of inner hair cells (IHCs) in adult mice and the overall transduction efficiency in murine cochlear OHCs decreases with age, regardless of the promoters used.^[^ ^20^, ^21^, ^22^, ^26^ ^]^ Several promoters, such as CBA, CAG, and CMV, have been used in vitro and in vivo for transgene expression in mammalian cells.^[^ ^27^ ^]^ These promoters have also been frequently used for transgene expression in cochlear sensory hair cells via injection to postnatal day 1–5 pups.^[^ ^28^, ^29^, ^30^, ^31^ ^]^ In this study, we have utilized the CMV promoter, one of the most used promotors.^[^ ^20^, ^21^, ^32^ ^]^

In contrast, IHCs do not exhibit significant changes in AAV transduction rate between neonatal and adult mice, showing high AAV transduction efficiency irrespective of age.^[^ ^33^, ^34^ ^]^ Except for their stereocilia and cuticular plate, OHCs and IHCs are immersed in perilymph,^[^ ^35^ ^]^ thus AAV injected via the semicircular canal into the endolymph or round window into the perilymph should reach IHCs and OHCs in adult mice. Other factors may also influence AAV transduction during maturation, such as increased cochlear fluid volume resulting in a relative decrease in the concentration of AAV particles reaching sensory hair cells or the ossification of the inner ear.^[^ ^8^ ^]^ Nevertheless, IHCs maintain high AAV transduction efficiency in adult mice that is similar to neonatal mice. This phenomenon indicates that some changes occur in OHCs specifically during inner ear maturation leading to decreased AAV transduction efficiency. The underlying mechanisms of this phenomenon remain unknown and enhancing AAV transduction efficiency in OHCs of adult mice remains a challenge.

AAV enters targeted cells through endocytosis in a receptor–dependent or ‐independent fashion. Receptors, such as heparan sulfate proteoglycan, FGF receptor, integrins αVβ, and sialic acids are reported to be necessary for AAV entry into targeted cell types.^[^ ^36^, ^37^, ^38^ ^]^ In 2016, through a haploid genetic screen, S. Pillay and colleagues found that AAV receptor (AAVR, *Kiaa0319l, Au040320*) serves as a critical receptor for AAV entry into multiple cell types, including human cell lines. Knockout or blockage of AAVR significantly reduces AAV infection in most serotypes, both in vivo and in vitro in abdominal tissue and cells.^[^ ^39^ ^]^ Their finding also suggests that the C‐tail of AAVR is necessary for formation of an AAV‐AAVR complex to endocytose into the trans‐Golgi network.

So far, there is no data describing the expression and distribution of AAVR in the inner ear, nor reports on the role of AAVR for AAV transduction in sensory hair cells. It is still unknown whether manipulation of AAVR expression could change AAV transduction efficiency in sensory hair cells. We hypothesize that the decrease in OHC transduction efficiency during maturation could be attributed to decreased expression of AAVR in cochlear OHCs. Enhancing the expression of AAVR in inner ear cells, including cochlear sensory hair cells and vestibular hair cells, should increase their sensitivity to AAV transduction. In this study, we first compared AAVR expression in neonatal and adult mice. Then, we measured the association between AAVR and AAV through co‐immunoprecipitation (co‐IP) and immunolabeling for co‐localization. Finally, we manipulated AAVR expression in vivo and in vitro with antibody inhibition, knock down, and conditional knock‐in of AAVR to study the transduction efficiency in cochlear sensory hair cells and vestibular hair cells with three vectors, AAV2, AAV2.7m8, and Anc80L65, that have been frequently used in inner ear research.

To our knowledge, our study is the first to demonstrate that AAVR serves as a receptor to promote AAV transduction efficiency in hair cells in the inner ear. Furthermore, our findings provide insights for future AAV‐mediated gene therapy for acquired and genetic hearing loss affecting OHCs in the organ of Corti, as well as for vestibular pathologies affecting the hair cells of the utricle and crista.

## 2 Results

### 2.1 There Is Decreased AAVR Expression in Inner Ear Hair Cells in Adult Mice Compared to Neonatal Mice

**Figure 1:**
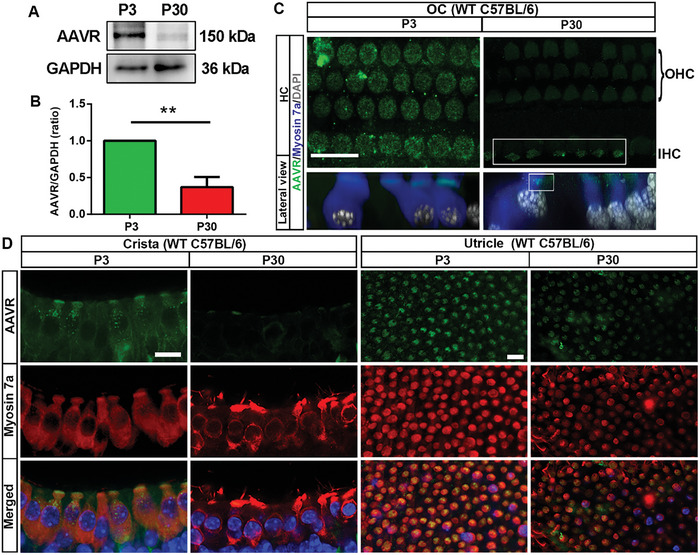
Expression of AAVR in inner ear hair cells is significantly decreased in adult mice compared to neonatal mice. A) Representative immunoblot images using whole inner ear homogenates of wild‐type C57BL/6 mice (WT) revealed a decreased AAVR band at 150 kDa at the age of 30 days (P30) compared to postnatal 3 days (P3). GAPDH (36 kDa) serves as the sample loading control. B) Semi‐quantification of AAVR band density confirmed a significant decrease at P30 compared to P3. Data are presented as means + SD, *n* = 3 per group with 4 ears pooled into one sample, **p < 0.01, analyzed by unpaired *t*‐test. C) Immunolabeling for AAVR (green) in the organ of Corti (OC) showed strong labeling in both IHCs and OHCs at P3. In contrast, AAVR labeling was weak in P30 mice with IHCs showing stronger labeling than OHCs, as outlined by the white box (upper panel). The lower panel shows reconstructed images from the side view, confirming that AAVR is localized in stereocilia of both IHCs and OHCs with strong labeling at P3. D) Immunolabeling for AAVR in the vestibular system (crista and utricle) also show a decrease in expression in hair cells at P30 compared to P3. Myosin 7a was co‐labeled to visualize sensory hair cells, and nuclei were stained with DAPI. Images in both C and D represent 3 samples at each age with scale bars = 10 µm.

First, we assessed AAVR expression in the mouse inner ear using Western blots and found that AAVR displayed a single band at 150 kDa in neonatal mice at 3 days of age (P3), but the band was much weaker at 30 days of age (P30) (**Figure** **1**A). Semi‐quantification of AAVR band intensity confirmed a significant decrease at P30 compared to P3 (Figure 1B, *t*_4_ = 7.896, *p* = 0.0014). Next, immunolabeling for AAVR on surface preparations of the organ of Corti (OC) and the crista and utricle of the vestibular system showed that AAVR was strongly expressed in the stereocilia region of both IHCs and OHCs at P3, whereas AAVR labeling was robustly decreased in OHCs at P30 (Figure 1C) with relatively stronger labeling in IHCs than OHCs (Figure 1C, highlighted by the white box). Reconstruction of Z‐sections confirmed that AAVR is localized to stereocilia of both IHCs and OHCs with strong labeling at P3 (Figure 1C, lower panel, side view). AAVR is also expressed in vestibular hair cells of the crista and utricle, with reduced labeling in P30 compared to P3 mice (Figure 1D). These results suggest that AAVR expression decreases in the inner ear of adult mice. Furthermore, we analyzed AAVR gene expression in OHCs, IHCs, Deiters’ cells, and pillar cells using single‐cell sequencing datasets from Dr. Ronna Hertzano's and Dr. David He's labs on gEAR (https://umgear.org/) (Figure S1, Supporting Information). The levels of AAVR mRNA in RiboTag OHCs (PrestinCre) were 2‐times higher at P8 than P14 and were stable from P14 until 10 weeks of age.^[^ ^40^ ^]^ The levels of AAVR mRNA by pipette and bulk‐sequenced pools of cells from CBA mice were significantly higher in IHCs than in OHCs at both ages of 1 and 9 months, while in pillar cells the levels were lower than in IHCs, and there was no significant difference between Deiters’ cells and IHCs at the age of 1 month.^[^ ^41^ ^]^ All these data from single‐cell RNA‐seq are consistent with our results.

### 2.2 The Efficiency of GFP Expression via AAV2 Transduction in Cochlear Outer Hair Cells and Vestibular Hair Cells Is Significantly Lower in Adult Mice than Neonatal Mice

Next, we compared GFP expression efficiency in cochlear and vestibular sensory hair cells with injection of the equal volume and concentration of AAV particles through the lateral semicircular canal (LSC) at P3 or P30. Since these three widely studied serotypes, AAV2, AAV2.7m8, and Anc80L65, were reported to have high transduction efficiency in sensory hair cells in neonatal mice,^[^ ^16^, ^18^ ^]^ we selected them for use in our study.

**Figure 2:**
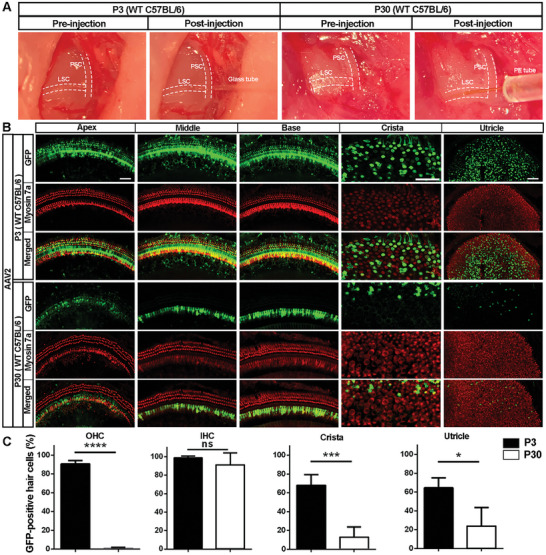
Efficiency of GFP expression in outer hair cells in the organ of Corti and vestibular hair cells in the crista and utricle via AAV2 transduction is significantly lower in adult mice than neonatal mice. A) Images indicate LSC micro‐injection of AAV vectors in neonatal and adult mice. PSC: posterior semicircular canal; LSC: lateral semicircular canal. B) Representative images revealed GFP expression in OHCs and IHCs in the apex, middle, and basal turns of the OC and in the vestibular hair cells of the crista and utricle of wild‐type C57BL/6 mice 20 days post AAV2 vector injection with the same number of viral particles via the lateral semicircular canal (LSC) either at P3 or P30. Hair cells were counter‐labeled with myosin 7a for visualization. Scale bar = 50 µm. C) Counting GFP‐positive OHCs in the OC and hair cells from the crista and utricle showed significantly lower GFP‐positive cells in P30 AAV‐injected mice compared to P3 AAV‐injected mice, while in IHCs there was no difference between P30 and P3 mice. *p < 0.05, ***p < 0.001, ****p < 0.0001, ns: not significant. *n* = 4 in each group, analyzed by unpaired *t*‐tests.

About 90% of OHCs and 98% of IHCs were GFP‐positive 20 days after AAV injection into neonatal mice at age P3. After AAV2 injection into P30 mice, however, the proportion of GFP‐positive OHCs dropped to only about 1%, whereas 91% of IHCs were positive (**Figure** **2**B,C). Statistical analysis confirmed a significant decrease in OHC transduction at P30 compared to P3 (Figure 2C, OHC, *t*_6_ = 47.28, *p* < 0.0001), but there was no significant difference in IHCs (Figure 2C, IHC, *t*_6_ = 1.1161, *p* = 0.2898). Additionally, there was 60% GFP‐positive HCs in both the crista and utricle in the P3 group, but this decreased to 12% and 23%, respectively, in the P30 group. Such a decrease was confirmed by statistical analysis (Figure 2C, crista: *t*_6_ = 6.988, *p* = 0.0004; utricle: *t*_6_ = 3.638, *p* = 0.0109). Similar results were also seen with injection of AAV2.7m8 or Anc80L65 serotypes (Figure S2, Supporting Information). Our results are consistent with other studies showing that sensory hair cells, including OHCs from the OC and hair cells from the crista and utricle of the vestibular system, in mature mice are less sensitive to AAV transduction compared to neonatal mice, with the exception of IHCs.^[^ ^20^ ^]^

### 2.3 AAV2 Viral Particles form Complexes In Vitro with AAVR

**Figure 3:**
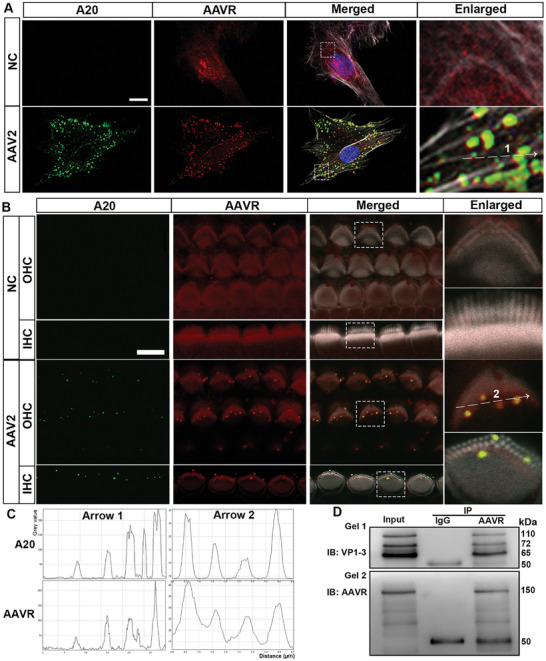
AAV2 viral particles form complexes with AAVR in in vitro models. Immunolabeling of intact AAV2 particles (A20, green) and AAVR (red) in A) HEI‐OC1 cells and B) P3 cochlear explants 20 min after AAV2 administration. Both HEI‐OC1 cells and stereocilia of IHC and OHCs revealed almost exact co‐localization of AAV2 and AAVR. The enlarged images from the white boxes are for better visualization. Arrows in the enlarged images indicate the axis for analysis of the plot profiles presented below (C). NC: negative control, blue: DAPI staining for nuclei; gray: phalloidin staining for hair cell structure. Scale bar = 10 µm. C) Intact AAV2 particle (A20) fluorescence and AAVR fluorescence signal plot profiles from the narrow band regions indicated by the arrows in the enlarged images (A and B) confirmed their colocalization in both HEI‐OC1 cells and hair cells of the cochlear explants. D) Formation of AAV2‐AAVR complexes was confirmed by co‐immunoprecipitation. Representative images with the first lane loaded with whole cell lysates as input, the second lane with a sample immunoprecipitated with an IgG antibody, and the third lane with a sample that was immunoprecipitated with an AAVR antibody. The membrane from gel 1 was immunoblotted with a VP1‐3 antibody and the membrane from gel 2 was immunoblotted with an AAVR antibody. The molecular weights of VP1‐3 are 110, 72, and 65 kDa and AAVR is 150 kDa. The 50 kDa band is from the IgG heavy chain from the secondary antibody.

The AAV2 vector directly integrates with AAVR during endocytosis in other systems in mice.^[^ ^39^, ^42^, ^43^ ^]^ To assess the interaction between AAV2 and AAVR in sensory hair cells, we performed immunohistochemistry and co‐immunoprecipitation (co‐IP) using HEI‐OC1 cells and cochlear explants. First, immunolabeling for intact AAV2 particles (detected by an A20 antibody) and AAVR in HEI‐OC1 cells showed dispersed attachment to the cytoskeleton on the cell surface 20 min after AAV2 administration and by 2 h after AAV2 infection, AAV2 distribution centralized to surround nuclei and co‐localized with AAVR (**Figure** **3**A). Such a distribution pattern is consistent with AAV2 infection in HeLa cells.^[^ ^44^ ^]^ Second, 20 min after AA2 administration into cochlear explants, AAV2 particles appeared around stereocilia bundles of OHCs and IHCs and co‐localized with AAVR (Figure 3B). Unlike in cell culture, they were not detectable 2 h after administration. A fluorescence signal plot profile illustrates a similar pattern of A20 and AAVR in HEI‐OC1 cells and cochlear explants, indicating co‐localization of AAV2 with AAVR (Figure 3C). Lastly, the formation of AAV2‐AAVR complexes was assessed by co‐IP experiments. Whole cell lysates (Input) or cell lysates immunoprecipitated with an AAVR antibody or IgG antibody to form pull‐down products were immunoblotted with an AAV2 capsid antibody (VP1‐3 capsid proteins, gel 1) or an AAVR antibody (gel 2). Three bands ranging from 65–110 kDa for VP1‐3 capsid proteins (Figure 3D, gel 1, 1st lane) or one at 150 kDa for AAVR (Figure 3D, gel 2, 1st lane) were detected in cell lysates. The VP1‐3 capsid proteins were also detected in the AAVR pull‐down product (Figure 3D, gel 1, 3rd lane) but not in IgG pull‐down controls (Figure 3D, gel 1, 2nd lane), indicating the formation of AAV‐AAVR complexes. The AAVR band was detectable in 3rd lane of gel 2 confirming successful pull‐down of the AAVR proteins. The band at 50 kDa was IgG heavy chain (gel 1 and 2).

### 2.4 Block of AAVR with Its Antibody Results in Decreased AAV2 Attachment and Diminished GFP Expression in HEI‐OC1 Cells and Cochlear Explants In Vitro

**Figure 4:**
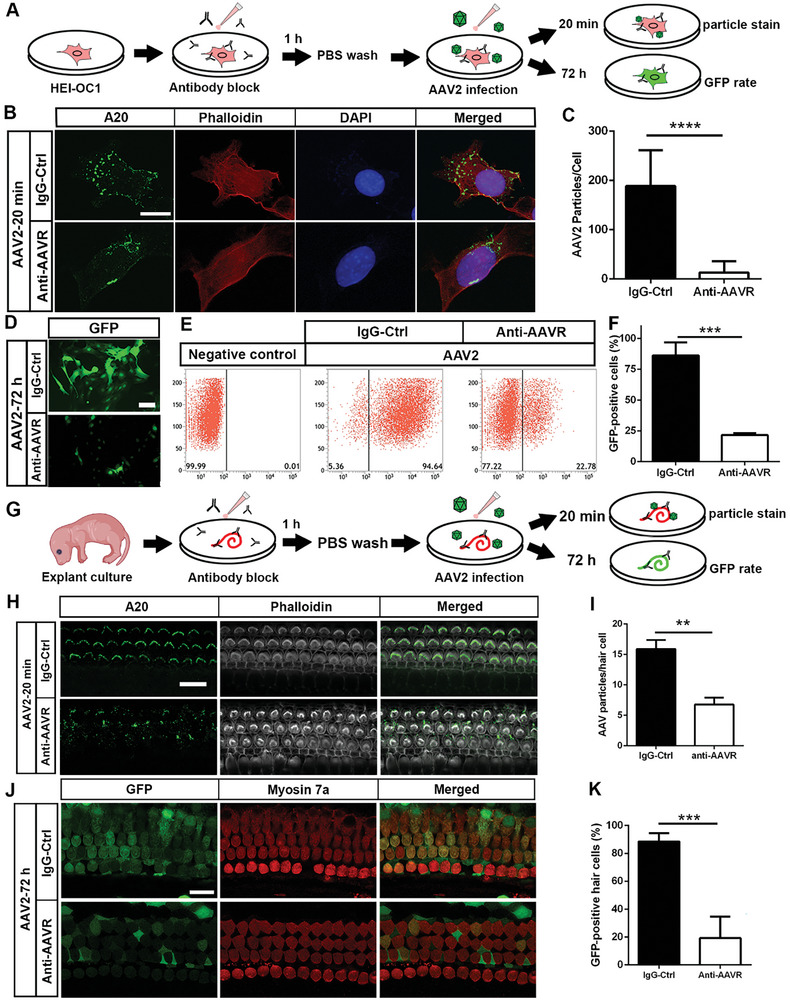
Blockage of AAVR by its antibody resulted in decreased AAV2 attachment and a lower GFP transduction rate in HEI‐OC1 and sensory hair cells of cochlear explants. A) The schematic diagram illustrates the antibody inhibition assay procedure in HEI‐OC1 cells. AAVR or IgG control antibody is applied to the media of cultured HEI‐OC1 cells for 1 h. Cells are then washed with prewarmed PBS before AAV2 administration. Samples are fixed 20 min after AAV2 exposure for the AAV attachment assay and 72 h after for GFP‐positive rate analysis. B) Representative immunolabeling of AAV2 particles (A20, green) showed fewer green dots on HEI‐OC1 cells in the AAVR antibody pre‐treated group compared to the IgG isotype control group. Scale bar = 10 µm. C) Quantification of AAV2 vector particles confirmed a significantly lower number in the AAVR antibody pre‐treated group compared to the control group 20 min after administration of AAV2. Data are presented as individual samples with means + SD, *n* = 12 in the IgG control group and *n* = 14 in the anti‐AAVR group from 3 independent experiments, ***p < 0.001, analyzed by unpaired *t*‐test. D) Representative fluorescence images showed significantly lower GFP‐positive cells in the AAVR antibody pre‐treated group compared to the control group 72 h post‐AAV2 vector administration. Scale bar = 25 µm. E) Flow cytometry analysis of the GFP‐positive cells showed lower infection efficiency with AAVR antibody pre‐treatment compared to the control group. A negative control (no AAV administration) was used for flow cytometry gate setting. F) Quantification of the percentage of GFP‐positive HEI‐OC1 cells confirmed a significant decrease in the AAV2 infection rate in the AAVR‐antibody blocked group; *n* = 3 in each group, ***p < 0.001, analyzed by unpaired *t*‐test. G) The schematic diagram illustrates the antibody inhibition assay procedure in cochlear explants. Equal amounts of AAVR antibody or IgG control are applied to cultured explants for 30 min. Then samples are washed with PBS before exposure to AAV2. Samples are then fixed 20 min after AAV application for the AAV attachment assay and 72 h after for GFP analysis. H) Pretreatment with an AAVR antibody reduced AAV attachment in sensory hair cells of neonatal cochlear explants. AAV particles were labeled by an A20 antibody (green) and hair cells were visualized by co‐staining with phalloidin. Scale bar = 10 µm. I) Quantification of AAV2 particles showed a significant reduction in the AAVR antibody pre‐treated group compared to the IgG control group. Data are presented as individual samples with means ± SD, *n* = 3 repetitions. **p < 0.01. J) Pre‐treatment with an AAVR antibody reduced GFP expression via AAV2 infection in the sensory hair cells of cochlear explants. Cochlear explants were co‐labeled with myosin 7a antibody for visualization of sensory hair cells. Scale bar = 10 µm. K) Quantification of GFP‐positive hair cells confirmed a significant decrease in the percentage of GFP‐positive cells with AAVR antibody pre‐treatment compared to an IgG control group. Data are presented as means + SD, *n* = 4 in each group. ***p < 0.001, analyzed by unpaired *t*‐test.

To determine the interaction of AAV2 with AAVR, we performed a pre‐antibody inhibition assay. First, HEI‐OC1 cells were pre‐treated with AAVR antibody for 1 h. The attachment of AAV2 particles (detected by immunolabeling for A20) was significantly decreased compared to an IgG isotype control group examined 20 min after viral vector administration (**Figure** **4**A–C, *t*_24_ = 8.612, *p* < 0.0001). Seventy‐two hours after AAV2 administration, GFP‐positive HEI‐OC1 cells were strongly decreased in the AAVR‐antibody pre‐treated group compared to controls (Figure 4D). Further, flow cytometry quantitative analysis showed an average of 86% GFP‐positive cells in the IgG control group, whereas only 21% were positive in the AAVR‐antibody pre‐treated group (Figure 4D,E), which was a statistically significant reduction (Figure 4F, *t*_4_ = 10.39, *p* = 0.0005).

Next, we assessed whether blocking AAVR reduces AAV2 infection in neonatal cochlear explants as illustrated in Figure 4G with 1‐h AAVR antibody pre‐treatment. Twenty minutes after AAV2 administration, the AAV2 particles were mainly attached to stereocilia bundles showing an upside‐down V shape in the IgG control group. In contrast, this distribution pattern was disrupted in AAVR‐antibody pre‐treated explants, which showed dispersed dots (Figure 4H). Quantification of AAV2 particle number showed a significant decrease in the AAVR pre‐treated group compared to the IgG controls (Figure 4I, *t*_24_ = 8.650, *p* < 0.0001). Furthermore, 72 h after AAV application, GFP expression in sensory hair cells was significantly decreased in the AAVR‐antibody pre‐treated group compared to IgG controls (Figure 4J). Quantification of GFP‐positive hair cells revealed a significant reduction in positivity rate from an average of 88% in the IgG control group to 19% in the AAVR‐antibody pre‐treated group (Figure 4K, *t*_6_ = 8.409, *p* = 0.0002). These results indicate that AAV2 physically interacts with AAVR.

### 2.5 Knockout of AAVR Blocks GFP Expression via AAV2 Transduction in Cochlear Sensory Hair cells In Vivo

**Figure 5:**
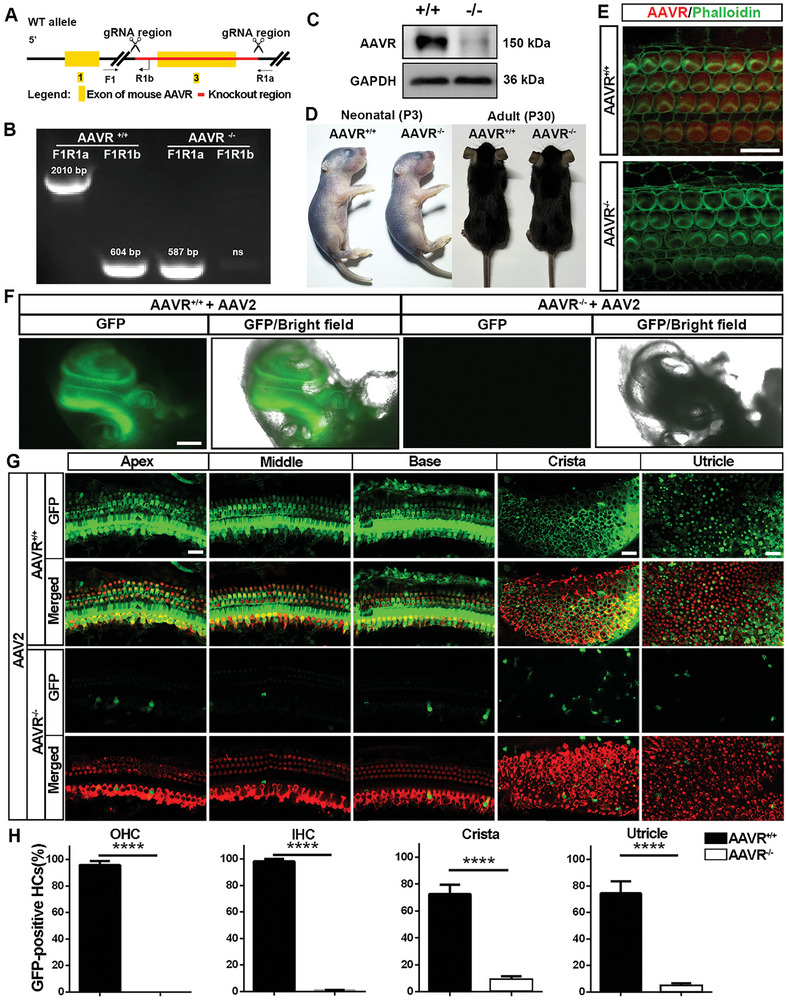
Knockout of AAVR blocks GFP expression via AAV2 transduction in cochlear hair cells in vivo. A) The design concept of AAVR knockout (KO) mice is shown in schematic form. Two guiding RNAs were generated to target deletion of exon 3 of AAVR. Forward primer 1 (F1) and two reverse primers (R1 and R2) were designed for genotyping. B) Representative PCR results from tail lysis showed a 2010‐bp band by F1R1 primer pairs and a 604‐bp band by F1R2 primer pairs in wild‐type (AAVR^+/+^) mice, a 587‐bp band by F1R1 primer pairs and no band by F1R2 primer pairs in homozygous AAVR KO mice (AAVR^−/−^). C) Immunoblots using whole inner ear homogenates from AAVR KO mice revealed strong reduction of AAVR band density at 150 kDa compared to wild‐type controls. GAPDH serves as the sample loading control. The experiment was repeated twice with 4 inner ears pooled into each sample. D) AAVR KO mice showed no obvious phenotypical differences compared to wild‐type littermates at neonatal (P3) and adult ages (P30). E) Immunolabeling for AAVR (red) on cochlear surface preparations of P3 AAVR KO mice confirmed knockdown of AAVR immunolabeling in IHCs and OHCs compared to wild‐type littermates. Hair cells were counterstained with phalloidin (green). Scale bar = 10 µm. F) There was no GFP (green) fluorescence in the cochleae of AAVR knockout mice, unlike wild‐type controls assessed 20 days after equal amounts of AAV2 vectors were injected through the LSC at P3. Images were taken by low‐power (4× lens) fluorescence microscopy with bright field images for visualization of cochlear structures. Scale bar = 0.5 mm. G) Immunolabeling for GFP expression (green) in IHCs and OHCs of the OC and the crista and utricle showed a sharp reduction in the AAVR knockout mice compared to wild‐type littermates. Hair cells were counter‐labeled with myosin 7a antibody. Scale bar = 10 µm. H) Quantification of GFP‐positive OHCs and IHCs in the OC and hair cells from the vestibular system (crista and utricle) confirmed a significant decrease. ****p < 0.0001, *n* = 4 in each group, analyzed by unpaired *t*‐tests.

We further used AAVR knockout (KO) mice to test whether AAVR is essential for AAV2 transduction in sensory hair cells. Based on a prior report showing that KO of AAVR significantly reduces AAV infection in the abdomen^[^ ^39^ ^]^ we generated AAVR KO mice using CRISPR‐based cleavage of exon 3 for functional destruction of AAVR in KO mice. Genotyping was performed using forward primer1 (F1), reverse primer1a (R1a), and reverse primer1b (R1b) to differentiate wild‐type and AAVR knockout alleles as illustrated in **Figure** **5**A. After PCR amplification of tail DNA, wild‐type littermates (AAVR^+/+^) showed a 2010‐bp band using the F1R1a primer pair and 604‐bp band with the F1R1b primer pair. AAVR KO (AAVR^−/−^) mice only generated a 587‐bp band with the F1R1a primer pair (Figure 5B). Moreover, the KO of AAVR was confirmed by immunoblots of inner ear homogenates at age P30 (Figure 5C). There was no obvious developmental or physical phenotype of AAVR KO mice compared to wild‐type littermates at neonatal and adult ages (Figure 5D), in agreement with a prior report.^[^ ^45^ ^]^ Knockout of AAVR was assessed in sensory hair cells by immunolabeling cochlear epithelia of neonatal mice at age P3. Immunolabeling for AAVR appeared in stereocilia of both IHCs and OHCs in wild‐type mice but was absent in AAVR KO mice assessed at P30 (Figure 5E). Auditory thresholds of AAVR‐KO mice at the age of 1 month were similar to their control littermates without significant shifts. Additionally, we did not observe signs of vestibular disturbance such as head tilting, circling, or disorientation in AAVR‐KO mice.

After successful generation of AAVR KO mice, we injected AAV2, AAV2.7m8, or Anc80L65 into the inner ear of both wild‐type and KO mice at age P3 via the LSC to assess whether AAVR is necessary for AAV transduction of GFP in sensory hair cells. Twenty days after AAV2 injection, the green fluorescence in the cochleae was easily visualized in wild‐type littermates (AAVR^+/+^) through low‐magnification fluorescent microscopy. In contrast, there was almost no detectable green fluorescence in KO mice (AAVR^−/−^) (Figure 5F). Cochlear surface preparations showed a significant decrease in GFP‐positive cochlear hair cells in the KO group compared to the wild‐type littermate group (Figure 5G), including OHCs (Figure 5H, *t*_5_ = 54.20, *p* < 0.0001) and IHCs (Figure 5H, *t*_5_ = 93.10, *p* < 0.0001) from the OC and vestibular hair cells from the crista (Figure 5H, *t*_5_ = 15.15, *p* < 0.0001) and utricle (Figure 5H, *t*_5_ = 12.47, *p* < 0.0001). Similar results were seen with AAV2.7m8 or Anc80L65 vector injection in AAVR KO mice and wild‐type littermates (Figure S3, Supporting Information). These results indicate that AAVR is a necessary factor for AAV vector transduction in sensory hair cells.

### 2.6 Conditional Overexpression of AAVR in Hair Cells Restores Their Sensitivity for AAV Transduction of GFP in Adult Mice

**Figure 6:**
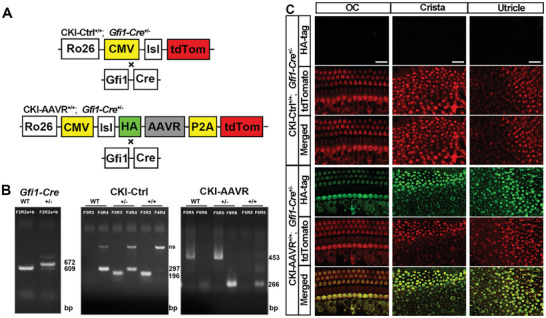
Design of conditional overexpression of AAVR in hair cells through cross with *Gfi1‐Cre* mice. A) The schematic diagram illustrates the design of AAVR conditional overexpression in sensory hair cells of mice. Briefly, conditional overexpression elements, including full‐length AAVR cDNA connected with an HA‐tag in the 5’‐end followed by a tdTomato sequence as a reporter, are separated from the CMV promoter by a loxP‐stop‐loxP (lsl) element. When crossed with *Gfi1‐Cre* ^+/−^ mice, the stop signal is removed to initiate exogenous AAVR translation and expression. Rosa26‐CMV‐lsl‐tdTomato mice were crossed with *Gfi1‐Cre* ^+/−^ mice to generate matched control mice. B) Representative genotyping PCR results of *Gfi1‐Cre* mice, CKI‐AAVR mice, and CKI‐Ctrl mice. Since *Gfi1‐Cre* homozygosity is lethal, only *Gfi1‐Cre* heterozygous mice were used in this study. The band for the wild‐type allele *Gfi1‐Cre* mice was detected at 609 bp and the mutant allele (±) at 672 bp by a F3R3 primer pair. The band for the WT allele of CKI‐Ctrl mice was detected at 297 bp by a F5R5 primer pair and the mutant allele at 196 bp by a F4R4 primer pair; the WT allele of CKI‐AAVR mice was detected at 453 bp by a F6R6 primer pair and the mutant allele at 266 bp by a F7R7 primer pair. C) Immunolabeling of HA‐tag (green) confirmed successful conditional overexpression of AAVR in sensory hair cells. Strong tdTomato expression (red) indicated the sufficient recombination of *Gfi1‐Cre* to the conditional overexpression of the AAVR sequence and matched controls in sensory hair cells from the organ of Corti, crista, and utricle. Scale bar = 10 µm.

**Figure 7:**
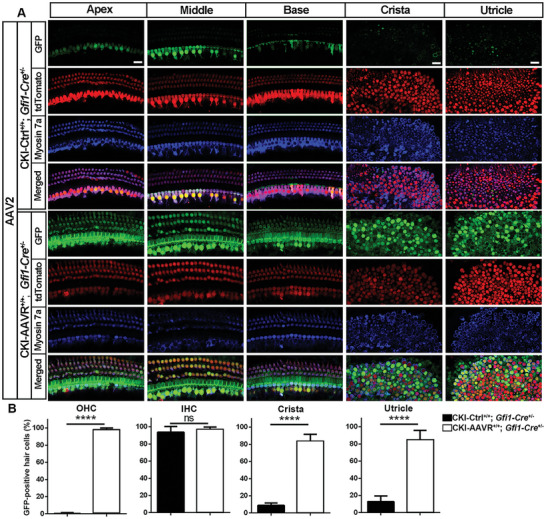
Conditional overexpression of AAVR in hair cells restores their sensitivity to AAV transduction in adult mice. A) AAV2 carrying a GFP reporter gene was injected through the LSC into CKI‐AAVR^+/+^; *Gfi1‐Cre* ^+/−^ and CKI‐Ctrl^+/+^; *Gfi1‐Cre* ^+/−^ mice at the age of P30. Twenty days post vector injection, GFP (green) expression was clearly seen in OHCs in the apical, middle, and basal turns of the OC and vestibular HCs of the crista and utricle in CKI‐AAVR^+/+^; *Gfi1‐Cre* ^+/−^ mice, but only weakly expressed in CKI‐Ctrl^+/+^; *Gfi1‐Cre* ^+/−^ mice. Red: tdTomato, blue: myosin‐7a‐labeled structure of HCs. Scale bar = 10 µm. B) Quantification of GFP‐positive hair cells confirmed a significant increase in transduction into OHCs in the OC and in vestibular HCs in the crista and the utricle. There was no difference in GFP expression in IHCs between the CKI‐AAVR^+/+^; *Gfi1‐Cre* ^+/−^ group and CKI‐Ctrl^+/+^; *Gfi1‐Cre* ^+/−^ mice. Data are presented as means + SD, *n* = 4 in each group. ****p < 0.0001, ns: non‐significant, analyzed by unpaired *t*‐tests.

Lastly, to determine whether overexpression of AAVR in sensory hair cells could restore their sensitivity to AAV transduction in adult mice, we generated conditional knock‐in of AAVR (CKI‐AAVR) in sensory hair cells by insertion of an AAVR conditional‐overexpression sequence into the Rosa26 locus, as illustrated in **Figure** **6**A. AAVR cDNA expression was controlled by a loxP‐stop‐loxP (lsl) switch and followed by the tdTomato reporter gene. Once crossed with *Gfi1‐Cre* mice, AAVR was able to be specifically expressed in sensory hair cells. Additionally, this AAVR sequence was tagged with an HA reporter sequence in the 5’‐end, used for detecting the conditional knock‐in of AAVR by immunolabeling of the HA‐tag. Appropriate conditional knock‐in control mice were generated by using CMV‐lsl‐tdTomato reporter mice crossed with *Gfi1‐Cre* mice. Since *Gfi1‐Cre* homozygous mice were reported to have IHC degeneration and develop early‐onset hearing loss^[^ ^46^, ^47^ ^]^ we only used *Gfi1‐Cre* heterozygous mice in this study. The genotyping of *Gfi1‐Cre*, CKI‐Ctrl, and CKI‐AAVR mice with different primer pairs showed wild‐type and mutant alleles (Figure 6B). Conditional AAVR knock‐in mice (CKI‐AAVR^+/+^; *Gfi1‐Cre* ^+/−^) and proper control mice (CKI‐Ctrl^+/+^; *Gfi1‐Cre* ^+/−^) displayed strong tdTomato signals in sensory hair cells, including OHCs and IHCs of the OC and vestibular hair cells of the crista and utricle (Figure 6C). These results indicate that *Gfi1‐Cre* drives recombination in almost all HCs, in agreement with a previous report.^[^ ^46^ ^]^ Moreover, labeling of the HA‐tag showed no fluorescence signal in the HCs of the control group, but strong signal in the HCs of conditional knock‐in mice, which further confirmed that the *Cre*‐driven recombination initiates expression of the inserted AAVR sequence (Figure 6C). Additionally, auditory thresholds of conditional AAVR knock‐in mice were similar to their respective control mice when measured at the age of 1 months and no signs of vestibular dysfunction were observed. After confirmation of conditional overexpression of AAVR in cochlear sensory hair cells, we injected AAV2, AAV2.7m8, or Anc80L68 vectors into AAVR conditional knock‐in (CKI‐AAVR^+/+^; *Gfi1‐Cre* ^+/−^) and proper control (CKI‐Ctrl^+/+^; *Gfi1‐Cre* ^+/−^) mice at age P30. Twenty days after injection, AAV transduction efficiency was detected by GFP‐positive sensory hair cells using surface preparations of the cochlear epithelium, crista, and utricle. Interestingly, conditional knock‐in of AAVR significantly increased GFP expression in almost all cochlear sensory hair cells and vestibular hair cells after AAV2 injection (**Figure** **7**A), whereas control mice only showed GFP expression in IHCs and a few vestibular hair cells. Counts of GFP‐positive OHCs revealed 95% in conditional knock‐in mice compared to no GFP‐positive OHCs in the control mice (Figure 7B, *t*_6_ = 98.78, *p* < 0.0001). There was no change in GFP‐positive IHCs between knock‐in and control mice (Figure 7B, *t*_6_ = 0.7399, *p* = 0.4873) as there were about 90% GFP‐positive IHCs on average in control mice. Vestibular GFP‐positive hair cells in the crista and utricle were also significantly increased from less than 20% in the control group to about 80% in the knock‐in group (Figure 7B, crista: *t*_6_ = 15.41, *p* < 0.0001; utricle: *t*_6_ = 11.74, *p* < 0.0001). Similar results were also seen after AAV2.7m8 and Anc80L65 injection in adult conditional knock‐in mice and control mice (Figures S4 and S5, Supporting Information). These results indicate that AAVR is essential for AAV vector transduction in hair cells in vivo.

## 3 Discussion

The key finding of this study is that the receptor AAVR is essential for AAV transduction in cochlear sensory hair cells and vestibular hair cells in mice. This is the first report that AAVR serves as a receptor for AAV particles entering sensory hair cells by forming a complex during endocytosis into hair cells. AAVR is expressed in the inner ear of both neonatal and adult mice, though to a significantly lesser extent in adult mice compared to neonatal mice, especially in cochlear OHCs. Inhibition of AAVR expression by pre‐treatment with its antibody in HEI‐OC1 cells or cochlear explants reduces AAV infection. Furthermore, knockdown of AAVR expression by administration of AAV vectors into the neonatal mouse inner ear significantly decreases GFP expression in sensory hair cells. In contrast, conditional knock‐in of AAVR in the inner ear restores sensitivity to AAV transduction of GFP in OHCs and vestibular hair cells after administration of AAV at adult ages.

Consistent with previous studies,^[^ ^20^, ^21^, ^22^ ^]^ our results show a significant decrease in GFP‐positivity rates in cochlear OHCs and vestibular HCs from the crista and utricle in adult mice compared to neonatal mice with the same titer and volume of AAV2, Anc80L65, or AAV2.7m8 injected into the inner ear. This could be associated with diminished AAVR expression in the inner ear of adult mice, as neonatal mice show a significantly higher amount of AAVR expression. In line with this notion, there is a relatively higher amount of AAVR expression in IHCs than OHCs with significantly higher AAV transduction efficiency in IHCs than OHCs in adult mice. Similarly, AAVR expression decreases in vestibular hair cells and AAV transduction efficiency of GFP is also low in hair cells of the crista and utricle in the mature inner ear. All these results strongly suggest that AAVR is an essential receptor for AAV infection in inner ear sensory hair cells. Additionally, analysis of a single‐cell sequencing dataset from Dr. Ronna Hertzano's lab using the RiboTag OHCs from the gEAR database (https://umgear.org/) is in line with our results, showing decreasing AAVR expression in OHCs during cochlear maturation. Furthermore, data from Dr. David He's lab collected from cochlear sensory hair cells of CBA mice by pipette and bulk‐sequenced pools of IHCs and OHCs at 1 and 9 months are also consistent with our data, showing that AAVR expression in IHCs is significantly higher than OHCs at P30. Although this study focuses on AAVR gene expression in sensory hair cells and does not evaluate AAVR expression in other cochlear cell types, such as Deiters’ and pillar cells, we recognize that supporting cells, stria vascularis cells, and neurons are important targets for gene therapy. Analysis of AAVR gene expression in Deiters’ and pillar cells from Dr. David He's lab's dataset shows no difference between Deiters’ cells and IHCs but less in pillar cells at the age of 1 month in CBA mice. However, we do not know why the expression of AAVR decreases in OHCs with maturation. This may be related to the regulatory mechanisms of AAVR in inner ear development, since maturation of the inner ear involves complex regulatory pathways.^[^ ^48^, ^49^ ^]^ The detailed mechanism of AAVR in regulation of inner ear development and its significance for normal hearing function needs further analysis.

Since AAVR is a transmembrane protein, using pre‐treatment with an antibody to block AAV infection efficiency is an ideal approach to confirm that AAV transduction occurs via AAVR expression.^[^ ^39^ ^]^ Our results show that blocking AAVR diminishes AAV2 attachment and reduces GFP expression in HEI‐OC1 cells and cultured cochlear explants. Consistent with our findings, pre‐treatment with AAVR antibody reduces AAV infection in airway epithelia and HeLa cells.^[^ ^39^, ^50^ ^]^ To confirm that AAV infection in sensory hair cells is associated with the AAVR receptor, we have generated AAVR knockout mice by removing the third exon in the AAVR cDNA. As AAVR is also expressed in the acrosome,^[^ ^51^ ^]^ knockout leads to infertility of homozygous male mice. Therefore, we have crossed AAVR‐KO heterozygous males with AAVR‐KO females to generate offspring in the expected Mendelian ratios. As we have predicted, knockout of AAVR significantly reduces GFP expression in sensory hair cells for all three assessed AAV vectors (AAV2, AAV2.7m8, and Anc80L65) after LSC injection into neonatal mice. Although there are literature reports that AAV2 is an AAVR‐dependent serotype in other tissues,^[^ ^39^, ^50^ ^]^ our data appears to be the first report that AAV2.7m8 or Anc80L65 transduction in sensory hair cells also depends on AAVR expression. Additionally, Mercedes Barzi et al. recently reported that a human liver chimeric transgene‐free mouse combined with liver humanized AAV receptor (AAVR) ablation, produces murine cells nonpermissive to AAV8 and AAV9 transduction.^[^ ^52^ ^]^ This suggests that AAV8 and AAV9 transduction also occurs in an AAVR‐dependent manner. Despite this, we do not rule out that other serotypes infecting sensory hair cells are independent of AAVR, as a distinct AAV capsid lineages, including AAV4 and AAVrh32.33, infect cells in the absence of AAVR.^[^ ^53^ ^]^ This may be related to how the AAV capsid enters cells. Prior to the identification of AAVR as an essential receptor for AAV‐mediated cell endocytosis, many serotypes that bind to distinct receptors on host cell surfaces had been identified. Some serotypes first attach to a primary receptor and then interact with a secondary receptor to facilitate viral entry. Identified primary receptors include heparan sulfate proteoglycans for AAV2, 3, and 6, *N*‐terminal galactose for AAV9, and specific N‐ or O‐linked sialic acid moieties for AAV1, 4, 5, and 6.^[^ ^54^ ^]^ Secondary receptors include fibroblast growth factor receptor (FGFR) and integrin for AAV2, hepatocyte growth factor receptor (c‐Met) for AAV2 and 3, and platelet‐derived growth factor for AAV5, although some of these are still in debate.^[^ ^55^, ^56^ ^]^ Recently, LY6A has been reported as a receptor for AAV‐PHP.eB transduction.^[^ ^57^ ^]^ However, no single receptor has been identified as common to all serotypes of AAV before identification of AAVR.^[^ ^39^, ^58^ ^]^ It is likely that AAVR plays a critical role due to its direct interaction with the AAV capsid to initiate the endocytosis process, while others serve as co‐receptors to assist the endocytosis process. Further studies are needed uncover these relationships. Our data supports that AAVR is the receptor for AAV entrance into hair cells in the inner ear through endocytosis. AAV and AAVR physically interact with each other, as evidenced by formation of complexes both in HEI‐OC1 cells and cultured cochlear explants. Moreover, AAV2 particles are colocalized with AAVR in the HEI‐OC1 cell cytoskeleton and on the cell surface or in stereocilia of cultured sensory hair cells 20 min after AAV application. These results suggest that AAVR serves as a carrier for AAV transport into the cytoplasm as previously reported.^[^ ^39^ ^]^

Finally, to further support our idea that AAVR is an essential receptor for AAV transduction in sensory hair cells, we have generated an AAVR conditional overexpression mouse by crossing loxP‐stop‐loxP controlled AAVR transgene mice with *Gfi1‐Cre* mice. As we expected, AAVR knock‐in not only restores the sensitivity of GFP expression in OHCs, but also in HCs from the crista and utricle to AAV2, AAV2.7m8, and Anc80L65 transduction after injection into adult mice. In line with our findings, Dr. James Zengel and colleagues developed a mouse model with *Cre*‐inducible AAVR overexpression, greatly increasing AAV transduction efficiency in diverse cell types, including muscle stem cells, which are typically refractory to AAV transduction.^[^ ^59^ ^]^ Similarly, Dr. Hiromi Sano et al. developed an AAVR expression model in targeted neural pathways, enabling efficient AAV vector‐mediated retrograde gene transfer.^[^ ^60^ ^]^ Although Gfi1‐Cre mice are a well‐accepted strain for conditional gene manipulation in sensory hair cells, potential limitations of Gfi1‐Cre mice include early‐onset hearing loss and off‐target recombination in other cell types, such as macrophages, as previously reported.^[^ ^46^ ^]^ For future study, other sensory‐hair‐cell‐specific Cre‐mice, such as Atoh1‐Cre or Prestin‐Cre, could be used as alternative vehicles for generation of conditional AAVR overexpression in sensory hair cells.^[^ ^61^, ^62^ ^]^ With regards to AAVR localization in the inner ear, our results show that AAVR is mainly expressed in the stereocilia of sensory hair cells. Given the fact that hair cell stereocilia bundles sit on the cuticular plate and are immersed in endolymph, injecting AAV into the endolymph may be expected to have high infection efficiency in sensory hair cells in neonatal and adult mice,^[^ ^14^, ^22^, ^63^ ^]^ although the relative position of the cochlear aqueduct changes with development and may also modulate distribution.^[^ ^64^ ^]^ Supporting the idea that AAVR localization influenced AAV infection is a study using human airway epithelia in which AAV has higher infection efficiency in the basolateral side of the cells where AAVR is localized.^[^ ^50^ ^]^

Current clinical trials using AAV‐mediated gene therapy for genetic hearing loss primarily target IHC mutations.^[^ ^1^, ^2^, ^3^ ^]^ Future developments in AAV‐mediated gene therapy may cover gene mutations in OHCs or in acquired hearing loss with OHCs as the main target. So far, it is not known whether AAVR is expressed in the human inner ear. Thus, detecting AAVR expression in donated human temporal bones or non‐human primates may provide insights into AAV sensitivity in the human inner ear. Although we have assessed three AAV serotypes in the AAVR knockout and AAVR conditional knock‐in mice, other serotypes need further investigation. Another limitation is the lack of data to clarify the mechanisms underlying decreased AAVR expression in sensory hair cells during maturation. Moreover, the genetically modified AAVR‐KO and AAVR‐CKI mice used in this study are on a C57BL/6 background, which carries the *Ahl* gene known to contribute to accelerated age‐related hearing loss.^[^ ^65^ ^]^ Due to this concern, we used AAVR‐KO and AAVR‐CKI mice at the age of 30 days, prior to any expected changes owed to the *Ahl* gene. AAVR‐KO and AAVR‐CKI mice showed normal ABR baseline thresholds compared to their littermates at the age of 30 days. Generally, the CBA strain is a well‐accepted mouse model for studies of acquired hearing loss due to its sensitivity to inner ear insults, such as noise‐ and ototoxic‐drug‐induced loss of sensory hair cells, and its lack of the *Ahl* gene variant.^[^ ^9^ ^]^ However, C57BL/6 mice are more commonly used for manipulation of gene expression given their well‐characterized genetics and availability of genetic tools. For studies on hearing function using C57BL/6 mice, replacement of the *Cadherin23‐753A* (*Ahl)* gene with the wild‐type variant *Cadherin23*‐753G from CBA/CaJ mice is essential to avoid the confounding factor of the *Ahl* gene, particularly for age‐related hearing loss studies and others utilizing mice at late ages. Replacement of *Ahl* with the wild‐type from the CBA/CaJ strain successfully prevents accelerated age‐related hearing loss.^[^ ^66^, ^67^ ^]^ So far, whether AAVR overexpression could enhance the infection efficiency of other viruses is not clear, nor is whether overexpression of AAVR may increase infection risk from other pathogens beyond mediating AAV endocytosis. However, studies have shown that AAVR overexpression does not alter the infection efficiency of adenovirus or lentivirus in HeLa cells.^[^ ^39^, ^68^ ^]^ AAVR mutations were originally found to be associated with dyslexia, which is potentially caused by alterations in neuronal migration.^[^ ^69^ ^]^ Further studies are needed to assess the safety of AAVR modulation in vivo.

In summary, our results clearly demonstrate that the receptor AAVR is essential for AAV transduction in cochlear sensory hair cells and vestibular hair cells in mice. Our findings also provide insight on AAV‐mediated inner ear gene therapy for targeting OHCs and vestibular hair cells. Additionally, our conditional AAVR knock‐in mice could serve as an ideal model for studying AAV‐mediated gene therapy for acquired hearing loss and late‐onset genetic hearing loss, as AAVR knock‐in significantly extends the AAV treatment window.

## 4 Experimental Section

### Animals

AAVR knockout (AAVR‐KO), tdTomato knock‐in control reporter mice (Rosa26‐CMV‐lsl‐tdTomato mice, CKI‐Ctrl), and AAVR conditional knock‐in (CKI‐AAVR) mice were generated by Cyagen and maintained on a C57BL/6J background at an animal facility of the Laboratory Animal Management Center of Sun Yat‐sen University. *Gfi1‐Cre* mice were kindly provided by Dr. Renjie Chai from Southeast University. Wild‐type C57BL/6 (WT) mice were purchased from Guang Dong Medical Laboratory Animal Center (GDMLAC, China). All mice had free access to water and a regular mouse diet (#GDMLAC‐260, GDMLAC, China) under a standard 12:12‐h light‐dark cycle with the room kept at ≈22 °C. Animal research protocols were approved by the Institutional Animal Care and Use Committee at Sun Yat‐sen University (#SYSU‐IACUC‐2023‐000892). Animal care and procedures were under the supervision of the Laboratory Animal Management Center of Sun Yat‐sen University.

### Extraction of Total Protein from Mouse Inner Ears

Mouse inner ears at the age of P3 and P30 were rapidly removed from the temporal bone and dissected to remove attached muscle and other soft tissues in pre‐cooled PBS, pH 7.4, containing a protease inhibitor (Sigma‐Aldrich, #P8340). To extract total protein from mouse inner ears, the tissues were homogenized in pre‐cooled RIPA lysis buffer (Sigma‐Aldrich, #R0278) with a protease inhibitor cocktail (Sigma‐Aldrich, #P8340) using a glass/glass micro‐tissue grind pestle and vessel for 5 min. After centrifugation at 12 000 × *g* at 4 °C for 10 min, the supernatants were collected as the total protein fraction. Four inner ears from two mice were pooled for one sample and stored at −80 °C.

### Western Blot Analysis

Protein samples (20 µg) from P3 or P30 mouse inner ear homogenates were separated by SDS‐PAGE. After electrophoresis, the proteins were transferred to a polyvinylidene fluoride (PVDF) membrane (Millipore, #05317) and blocked with 5% albumin (Sigma‐Aldrich, #A7906) in phosphate buffered saline (PBS) containing 0.1% Tween‐20 (PBS‐T) (Sigma‐Aldrich, #P1379) at room temperature for 30 min. The membranes were incubated with rabbit monoclonal anti‐AAVR (1:1,000, Abcam, #315028) or anti‐GAPDH (1:5 000, Millipore, #ABS16) at 4 °C overnight. Then, the PVDF membranes were washed with PBS‐T and incubated with an appropriate secondary antibody (Cell signaling technology, #7074) at a concentration of 1:3000 for 1 h at room temperature. Following extensive washing of the membrane with PBS‐T, the bands were visualized by chemiluminescent Western blot reagent (Thermofisher Scientific, #34075). The band densities were measured using ImageJ software and the AAVR/GAPDH ratio was calculated from the band densities run on the same gel to normalize for differences in protein loading. Finally, the difference in the ratio of the control and experimental bands was tested for statistical significance comparing P3 versus P30.

### Immunocytochemistry for Cochlear Surface Preparations

The surface preparation and sample mounting techniques were identical to those described in the previous publication.^[^ ^7^ ^]^ Briefly, the samples were fixed with 4% paraformaldehyde (PFA) at 4 °C overnight, and then permeabilized with PBS containing 1% Triton‐X (Sigma Aldrich, #T8787) at room temperature for 30 min. After blocking with 10% goat serum, the specimens were incubated with primary antibodies: mouse polyclonal anti‐AAVR at 1:200 (Abcam, #ab105385) and polyclonal rabbit anti‐Myosin 7a (Proteus Biosciences, #25‐6790) at 1:500 in darkness at 4 °C for 24 h. They were then incubated with the Alexa‐Fluor‐594‐ or ‐488‐conjugated secondary antibody (Cell Signaling Technology, #8889 or #4408) at a concentration of 1:200 at 4 °C overnight. Nuclei were counterstained with DAPI (1:1000, Cell Signaling Technology, #4083) at room temperature for 10 min. Immunolabeled images were taken with a Zeiss LSM 880.

### Immuno‐Colocalization of AAV2 with AAVR in HEI‐OC1 Cells and Cochlear Explants

HEI‐OC1 cells, a well‐characterized inner ear cell line, or cochlear explants were prepared as per prior publications.^[^ ^70^, ^71^, ^72^, ^73^ ^]^ Briefly, HEI‐OC1 cells were cultured in 6‐well plates at a density of 1 × 10^5^ cells per well in Dulbecco's modified Eagle's medium (DMEM, Gibco, #11965092) supplemented with 10% fetal bovine serum (FBS, Gibco, #A5670701) in a humidified incubator set to 33 °C with 5% CO_2_ and 95% humidity overnight before exposure to AAV. The explants were cultured in 1× basal Eagle's medium (Sigma Aldrich, #B9638) containing 1% bovine serum albumin (Biofroxx, #10GR100) and 1% insulin transferrin selenium (Gibco, #51500056) at 37 °C in a humidified incubator with 5% CO_2_ overnight before AAV administration. AAV2 vectors were diluted into culture medium and incubated with HEI‐OC1 cells or cochlear explants at a final concentration of 5 × 10^9 ^GC mL^−1^ for 20 min in a humidified incubator. The culture medium was then removed, and the cells or explants were washed with PBS before fixation with 4% PFA in room temperature for 1 h. Note that such fixation should not exceed 1.5 h to preserve AAV2 antigen specificity. After 1% PBS‐T permeabilization and 10% goat serum blockage, the specimens were incubated with rabbit monoclonal anti‐AAVR at 1:200 (Abcam, #ab315028) and monoclonal mouse anti‐AAV2 (Progen, #61055) at 1:20 in darkness at 4 °C for 24 h. They were then incubated with the Alexa‐Fluor‐594‐ or ‐488‐conjugated secondary antibody (Cell Signaling Technology, #8889 or #4408) at a concentration of 1:200 at 4 °C overnight. Finally, samples were counterstained with phalloidin‐633 (Thermofisher, #A22284) and DAPI (1:1000, Cell Signaling Technology, #4083) at room temperature for 10 min (Cell Signaling Technology, #4083). Immunolabeled images were taken with a Zeiss LSM 880. The colocalization between AAV2 particles and AAVR was analyzed via ImageJ software by generating plot profiles in two channels.

### AAV2‐AAVR Co‐Immunoprecipitation from HEI‐OC1 Cells

AAV2 was diluted in the culture medium of HEI‐OC1 cells at 2 × 10^10 ^GC mL^−1^ and incubated at 37 °C for 20 min. The control group was treated with same volume of PBS. After washing three times with pre‐warmed PBS, cells were collected by centrifuging at 800 × *g* for 3 min at 4 °C. Cell pellets were washed with pre‐cooled PBS, followed by treatment with the commercial co‐IP kit as per the company's instructions below (Sangon Biotech, #C600689). One milliliter of lysis buffer was added to the cell pellet and homogenized with a glass homogenizer for 30 strokes, then centrifuged at 12 000 rpm for 5 min and the supernatant was collected as the cell lysate. Next, 0.2 mL of lysate was taken as the input sample and 0.5 mL of cell lysate was incubated with 1 µg anti‐AAVR antibody or non‐specific IgG control antibody at 4 °C on a static flat platform overnight. After incubation, the cell lysates were transferred to the Protein‐A/G beads in the spin column and incubated at 4 °C overnight. Then the column was inserted into 2‐mL microcentrifuge tubes and spun at 12 000 × *g* for 30 s at 4 °C, washing the beads six times with 700 µL of IP buffer and one time with 10% diluted IP buffer. To the beads was added 40 µL of loading buffer and they were mixed gently before heating the samples at 95 °C for 5 min. Finally, after the column was centrifuged at 12 000 × *g* for 30 s, the eluted immunoprecipitate was collected for SDS‐PAGE. The input samples, IgG control (Abcam, #ab172730), and anti‐AAVR (Abcam, #ab315027) pulldown groups were immunoblotted with anti‐VP1‐3 and anti‐AAVR antibodies to verify the AAV‐AAVR interaction.

### AAVR Antibody Pre‐Treatment to Block AAVR in HEI‐CO1 and Cochlear Explants

HEI‐OC1 cells or cochlear explants were cultured overnight as described above. Based on a report,^[^ ^39^ ^]^ HEI‐OC1 cells or cochlear explants were incubated with rabbit monoclonal anti‐AAVR antibody (Abcam, #ab315028) or an IgG isotype control (Abcam, #ab172730) at a concentration of 50 µg mL^−1^ in medium for 1 h at 4 °C. Following incubation, the HEI‐OC1 cells or cochlear explants were gently washed three times with PBS and then switched to fresh, antibody‐free culture medium. The cells or cochlear explants were subsequently infected with AAV2 at a concentration of 5 × 10^10^ GC mL^−1^ diluted in culture medium and incubated for either 20 min or 72 h in a humidified incubator (HEI‐OC1 cells: 33 °C; explants: 37 °C) with 5% CO_2_ and 95% humidity. After the 20‐min AAV2 incubation, the culture medium was discarded, and the cells or explants were washed with pre‐warmed PBS before being fixed with 4% PFA at room temperature for 1 h. The amount of AAV2 particle attachment was then measured through immunolabeling of the intact AAV capsid using the A20 antibody (1:20, Progen, #61055) and counterstaining with phalloidin‐594 (1:200, Thermofisher, #A12381) to visualize HEI‐OC1 cell or sensory hair cell structures. DAPI was used to stain HEI‐OC1 cell nuclei (1:1000, Cell Signaling Technology, #4083). Seventy‐two hours post AAV2 incubation, the HEI‐OC1 cells were trypsinized (Gibco, #25200072), resuspended in PBS, and analyzed for green fluorescent protein (GFP) expression via flow cytometry. A negative control group without AAV2 infection was utilized to set the gate threshold for GFP quantification. Seventy‐two hours post AAV2 infection, cochlear explants were counter‐labeled with Myosin 7a (Proteus Biosciences, #25‐6790) for sensory hair cells to count the GFP‐positive hair cells in the middle turn with a 40× magnification lens using a Zeiss LSM 880 microscope.

### Genotyping

DNA extracted from tail samples was used to genotype mice. The program for PCR included initial denaturation at 94 °C for 5 min followed by 35 cycles for 30‐s denaturation at 94 °C, 45‐s annealing at 58 °C, 45‐s extensions at 72 °C, and a final extension at 72 °C for 10 min. The PCR primers used to identify the AAVR‐KO allele were F1 (5’‐GCA AAA TCA TGT GAC TGG CAA ACA‐3’), R1a (5’‐CTG GTT TGG ACT TGA CAG AGA CAA‐3’), and R1b (5’‐GGC AGG TTT AGG GTA AGA AAA‐3’). The primers used to identify the *Gfi1‐Cre* allele were F2 (5’‐GCC CAA ATG TTG CTG GAT AGT‐3’), R2a (5’‐GGG ATA ACG GAC CAG TTG‐3’), and R2b (5’‐CCG AGG GGC GTT AGG ATA‐3’). To identify CKI‐Ctrl mice, the F4 (5’‐AAG GGA GCT GCA GTG GAG TA‐3’) and R4 (5’‐CCG AAA ATC TGT GGG AAG TC‐3’) primers were used. The F3 (5’‐GGC ATT AAA GCA GCG TAT CC‐3’) and R3 (5’‐CTG TTC CTG TAC GGC ATG G‐3’) primers were used to identify the CKI‐Ctrl mutant allele. For CKI‐AAVR genotyping, F5 (5’‐CAC TTG CTC TCC CAA AGT CGC TC‐3’) and R5 (5’‐ATA CTC CGA GGC GGA TCA CAA‐3’) primers were used to identify the CKI‐AAVR wild‐type allele, F6 (5’‐TCA ATC CAG CGG ACC TTC CTT‐3’) and R6 (5’‐CTT TAT TAG CCA GAA GTC AGA TGC‐3’) were used to identify the CKI‐AAVR mutant allele. The PCR products were separated by a 2% agarose gel and the genotypes of mice were determined based on the base‐pair size of detected bands.

### Delivery of an AAV Vector into the Inner Ear of Postnatal Day 3 Mice via the Lateral Semicircular Canal

AAV2, AAV2.7m8, and Anc80L65 serotypes were purchased from VectorBuilder at a concentration of 5 × 10¹^2^ GC mL^−1^ and stored at −80 °C prior to use. All vectors contained a GFP reporter gene driven by a CMV promoter. Vectors were injected into P3 pups via the lateral semicircular canal (LSC) using beveled glass microinjection pipettes. Pipettes were pulled from capillary glass (WPI, Sarasota, FL) using a P‐2000 pipette puller (Sutter Instrument, Novato, CA). Wild‐type or AAVR‐KO P3 pups were anesthetized by inducing hypothermia in ice water for ≈2 min until loss of consciousness, then placed on an ice‐cooled platform for less than 10 min during the surgery. After disinfecting the area with 70% ethanol, a 0.5‐cm retroauricular incision was made to expose the LSC, and the glass micropipette tip was advanced into the canal. One microliter of virus was injected over 5 min using a Nanoliter Injector controlled by a MICRO4 controller (World Precision Instruments). After injection, the skin incision was closed with 5–0 nylon sutures. The pups were allowed to recover from anesthesia on a warmed pad (≈36 °C) for about 5 min before being returned to their mothers to be nursed until weaning.

### Delivery of an AAV Vector into the Inner Ear of Adult Mice via the Lateral Semicircular Canal

Wild‐type, CKI‐Ctrl, and CKI‐AAVR adult (P30) mice on C57/BL6 background were used for AAV injection. Anesthesia was induced through intraperitoneal (IP) injection of a mixture of ketamine (100 mg kg^−1^) and xylazine (10 mg kg^−1^). Mouse body temperature was maintained near 37 °C with a heating pad. AAV delivery was done using the LSC approach. Briefly, after shaving and sterilizing the area, a 1‐cm post‐auricular incision was made using surgical scissors. Then the sternocleidomastoid muscle was bluntly dissected to expose the LSC and a 27‐gauge needle was used to drill into the lumen of the LSC. After confirmation that the LSC lumen was successfully accessed by lymphatic leakage, a microinjection system (World Precision Instruments, Nanoliter2000) was used in conjunction with a polyethylene tube (0.0065‐in outer diameter, Nordson Medical, #141‐0002) connected to a pulled glass micropipette (World Precision Instruments, #PB150F‐4). The tip of the polyethylene tube was inserted into the LSC to around a 2‐mm depth. Tissue adhesive (World Precision Instruments, #7342) was used to seal the aperture between the inserted tube and the LSC to prevent lymphatic leakage from the injection site before injection of viral vector. One microliter of AAV2, AAV2.7m8, or Anc80L65 at a concentration of 5 × 10^12^ GC mL^−1^ was injected into the LSC at a speed of 200 nL min^−1^. After injection, the LSC fenestration was closed over with nearby muscle and double sealed with tissue adhesive. Finally, the incision was closed with 5–0 nylon sutures.

### Statistical Analyses

Data were analyzed using GraphPad 5.0 software for Windows. Biological sample sizes were determined based on the variability of measurements, the magnitude of differences between groups, and experience from the previous studies, with stringent assessments of difference. Differences between two groups were evaluated using two‐tailed unpaired *t*‐tests. A *p*‐value of < 0.05 was considered statistically significant. Data are presented as means ± SD. Sample sizes are indicated for each figure. Detailed statistical values are summarized in Tables S1–S3 (Supporting Information).

## Conflict of Interest

The authors declare no conflict of interest.

## Author Contributions

F.W., G.C., and R.H. contributed equally to this work. F.W., Z.H., and Y.Z. designed experiments. F.W., G.C., R.H., P.L., J.L., and W.Z. performed research; F.W., G.C., and R.H. analyzed data; F.W. and S.S. wrote the manuscript. All authors have reviewed the contents of the manuscript, approve of its contents, and validate the accuracy of the data.

## Supporting information



Supporting Information

## Data Availability

The data that support the findings of this study are available from the corresponding author upon reasonable request.
