# Supplementary material for: AAVR Expression is Essential for AAV Vector Transduction in Sensory Hair Cells
Source: Adv Sci (Weinh). 2025 Jan 7;12(29):2408873. doi: 10.1002/advs.202408873 (PMC12362819; doi:10.1002/advs.202408873)
Supplement: Supplementary file 1 — Supporting Information [file ADVS-12-2408873-s001.docx]

**Supplementary materials:**

**
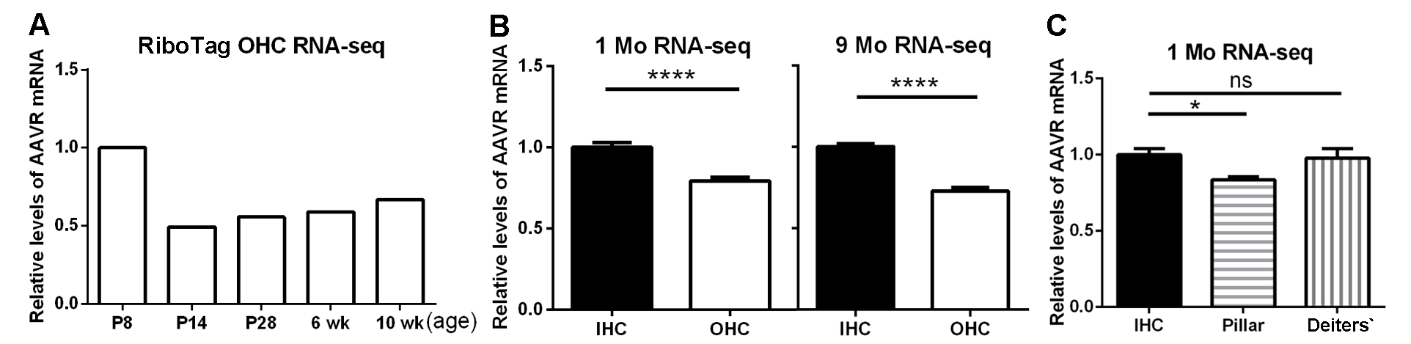
**

**Figure S1. AAVR mRNA levels are analyzed from single-cell RNA-seq data from the gEAR database of Dr. Hertzano’s and Dr. He’s labs’ datasets.**

**A.** Translation levels of AAVR mRNA in OHCs is about 2-times higher at P8 than P14 and is stable from P14 until 10 weeks of age. This data is analyzed from Dr. Hertzano’s dataset, and each sample was collected from 5 mice (10 cochleae) (Chessum et al.,2018).

**B.** Analysis from Dr. David He’ s dataset shows that the levels of AAVR are significantly higher in IHCs than OHCs at both 1 month and 9 months of age (Li et al., 2018). For the 1-month group: *n* = 4 repetitions for IHCs and *n* = 7 for OHCs; 9-months: *n* = 4 for IHCs and *n* = 4 for OHCs, each repetition consisting of a collection of 1000 cells; *****p* < 0.0001, analyzed by unpaired *t*-test. Data are presented as means + SD.

**C.** Analysis from Dr. David He’ s dataset shows that the levels of AAVR mRNA in IHCs are higher than in pillar cells, but there is no significant difference between IHCs and Deiters’ cells at the age of 1 month. Data are presented as means + SD, *n* = 3 repetitions with each cell type, each repetition a collection of 1000 cells, **p* < 0.05, analyzed by unpaired *t*-tests.


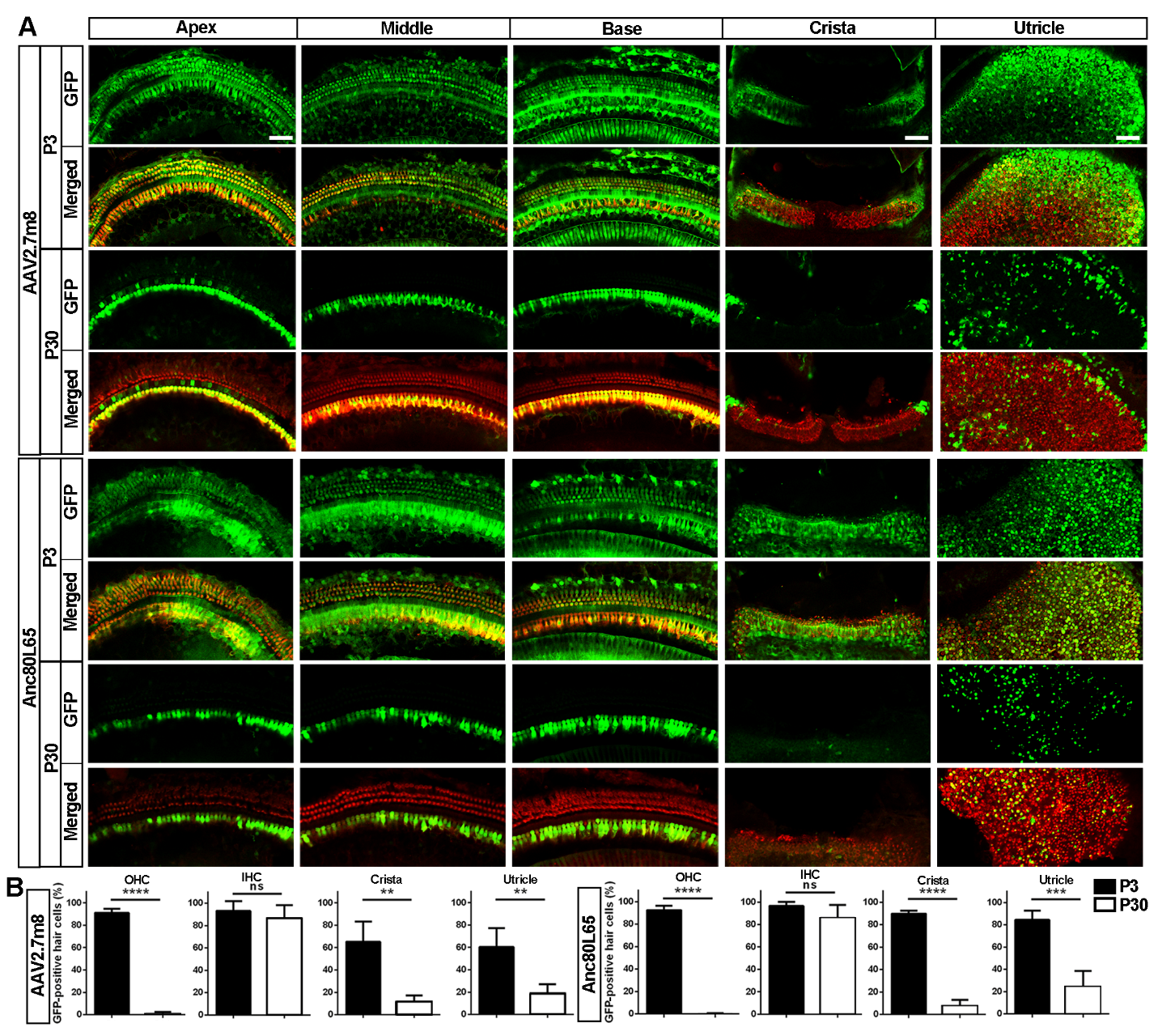


**Figure S2. Efficiency of AAV2.7m8 transduction in OHCs of the organ of Corti and vestibular HCs of the crista and utricle is significantly lower in adult mice than neonatal mice.**

**A.** Representative images show GFP expression in IHCs and OHCs from the apex, middle, and basal turns of the OC and and vestibular hair cells of the crista and utricle 20 d after injection of equal amounts of AAV2,7m8 or Anc80L65 vectors via the LSC at the age of P3 or P30. Hair cells were visualized with myosin 7a (red) antibody labeling. Scale bar = 30 μm.

**B.** Quantification of GFP-positive OHCs in the OC and vestibular HCs of the crista and utricle of AAV-injected mice showed significantly lower GFP-positive hair cells at the age of P30 than at P3. However, the transduction rate of IHCs was not significantly different between the two age groups. Data are presented as means + SD, *n* = 4 in each group, ***p* < 0.01, ****p* < 0.001, *****p* < 0.0001, ns: not significant; analyzed by unpaired *t*-tests. Detailed statistical values are listed in table S1.


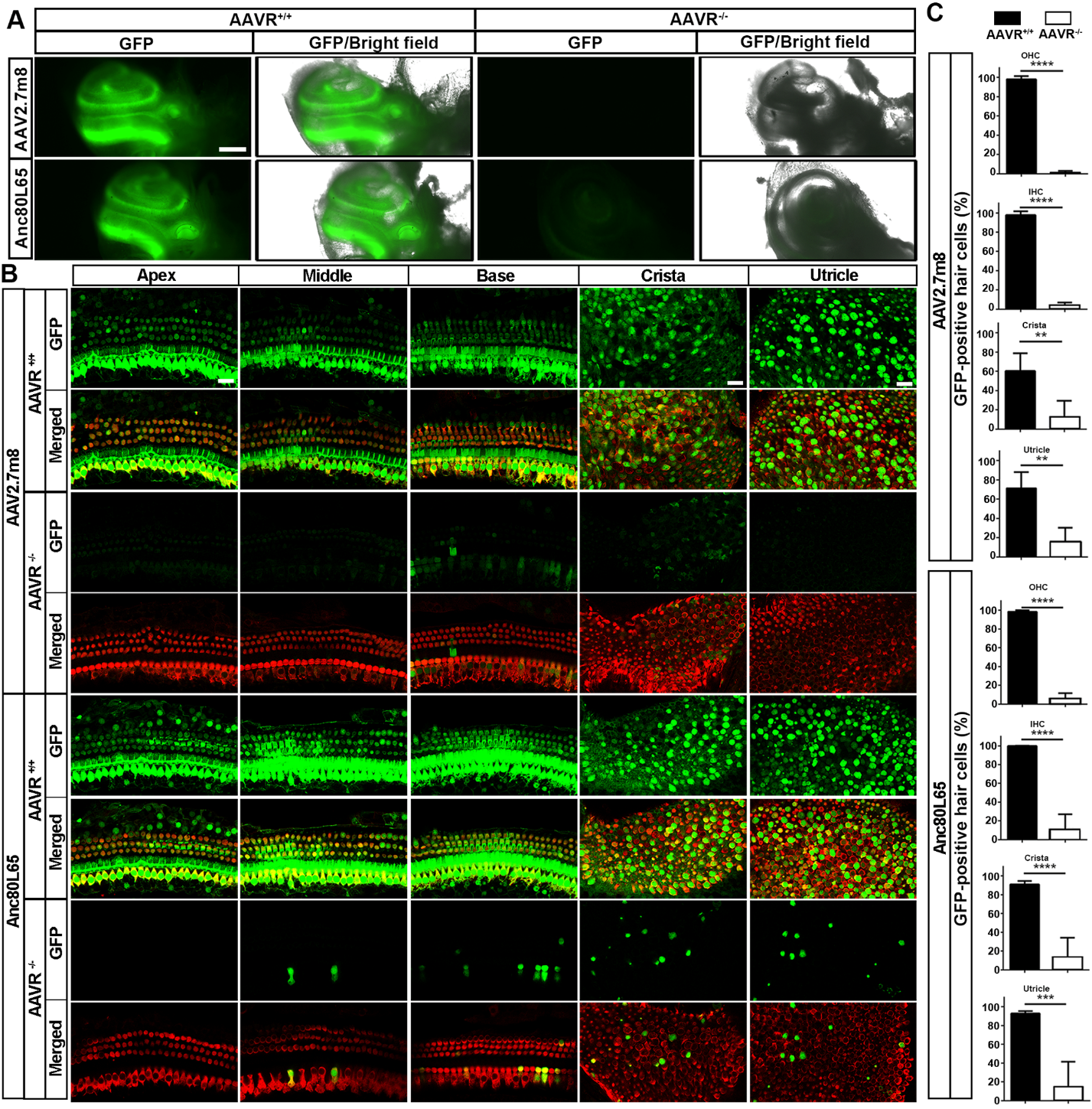


**Figure S3. Knockout of AAVR inhibits GFP expression via AAV2.7m8 and Anc80L65 transduction in cochlear and vestibular hair cells *in vivo.***

**A.** While robust transduction of GFP (green) is seen in wild-type controls, there was almost no GFP fluorescence in AAVR knockout mice 20 d after AAV2.7m8 and Anc80L65 injection at the age of P3. Images were taken using a 4× lens fluorescence microscope. Bright-field images were taken to visualize cochlear structures. Scale bar = 0.5 mm.

**B.** GFP expression in OHCs and IHCs in the apical, middle, and basal turns of the OC and vestibular HCs of the crista and utricle was greatly decreased in AAVR knockout mice compared to the wild-type control group. Hair cells were labeled by myosin 7a antibody 20 d after AAV2.7m8 or Anc80L65 injection. Scale bar = 10 µm.

**C.** Quantification of GFP-positive hair cells confirmed a significant decrease in AAV2.7m8 and Anc80L65 transduction into OHCs and IHCs in the OC and vestibular HCs of the crista and utricle. Data are presented as means + SD, AAV2.7m8: *n* = 4 in AAVR^+/+^ group and *n* = 5 in AAVR^-/-^ group; Anc80L65: *n* = 5 in AAVR^+/+^ group and *n* = 4 in AAVR^-/-^ group. ***p* < 0.01, ****p* < 0.01, *****p* < 0.0001, analyzed by unpaired *t*-tests. Detailed statistical values are listed below in table S2.


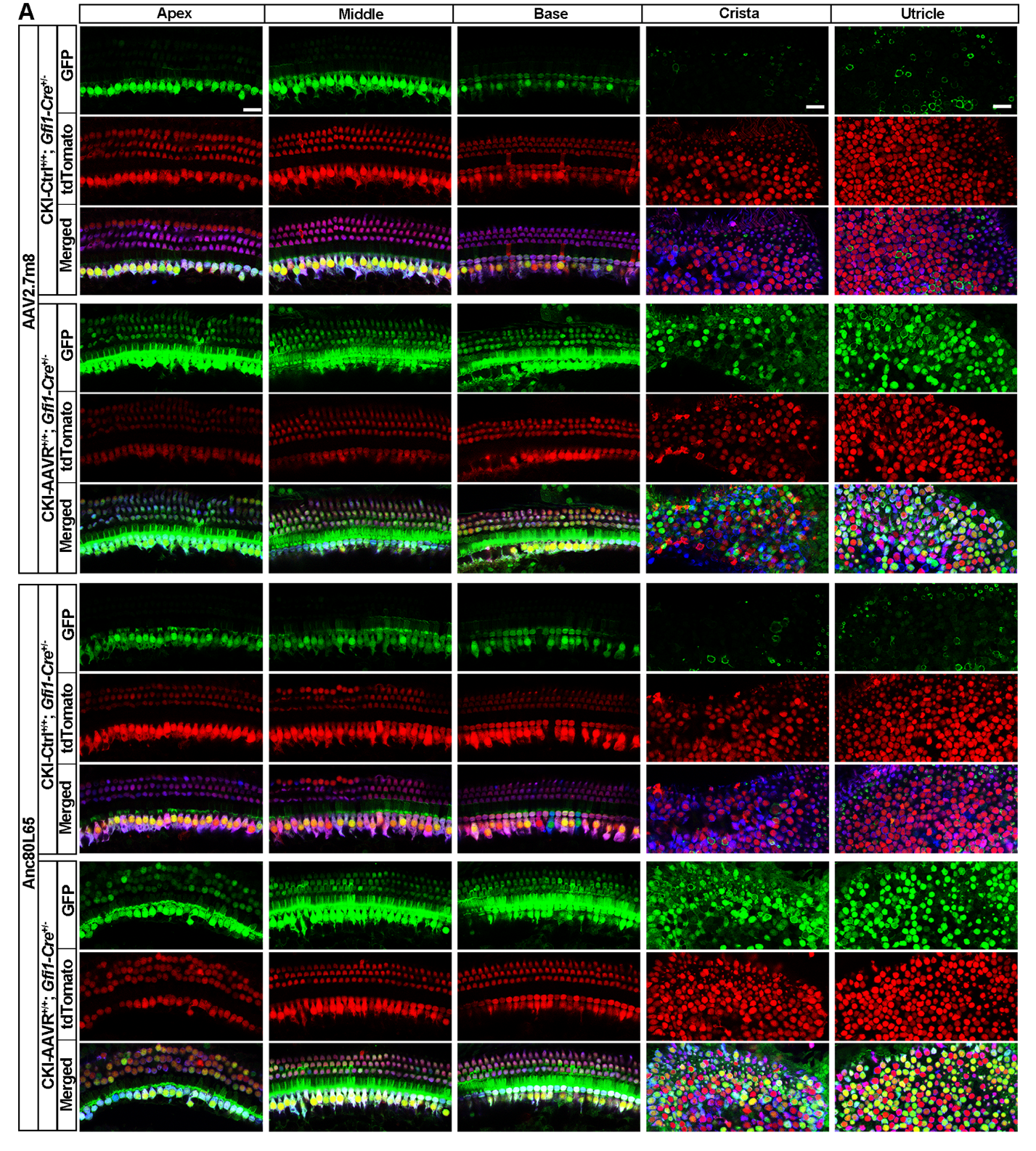


**Figure S4. Conditional knock-in of AAVR in hair cells restores their sensitivity to AAV2.7m8 and Anc80L65 transduction in adult mice.**

GFP-positive hair cells in cochlear surface preparations revealed that overexpression of AAVR in inner ear hair cells restored AAV2.7m8 and Anc80L65 transduction efficiency in adult mice. Samples were counter-labeled with myosin 7a (blue) for visualization of sensory hair cell structures. Scale bar = 10 µm.


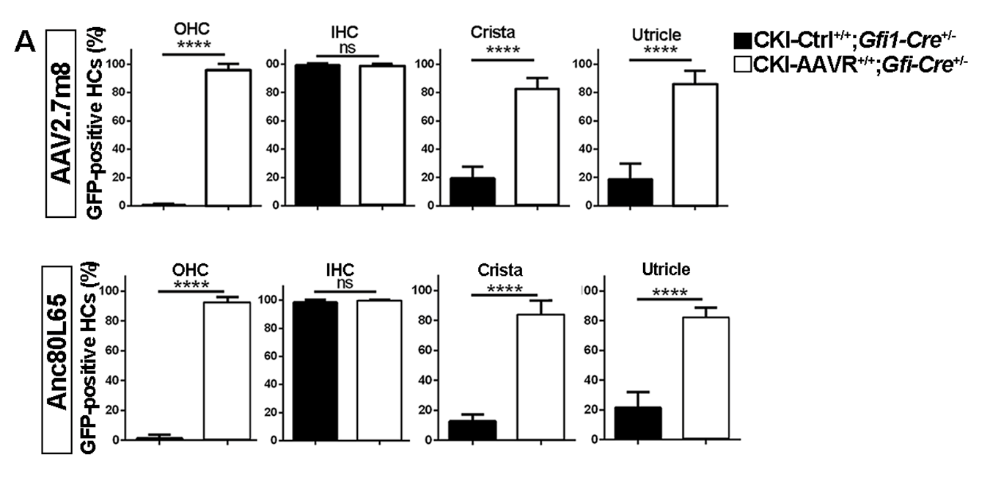


**Figure S5. Statistical analysis confirmed that conditional knock-in of AAVR in hair cells restores their sensitivity to AAV2.7m8 and Anc80L65 transduction in adult mice.**

Quantification of GFP-positive hair cells confirmed a significant increase in transduction efficiency in OHCs of the OC and vestibular HCs of the crista and utricle. GFP transduction in IHCs was not significantly changed by the conditional AAVR knock-in (CKI-AAVR^+/+^; *Gfi1-Cre*^+/-^) mice compared to the control mice (CKI-Ctrl^+/+^; *Gfi1-Cre*^+/-^); *n* = 4 in each group, *****p* < 0.0001, ns: not significant, analyzed by unpaired *t*-tests. Detailed statistical values are listed below in table S3.

**Tables:**


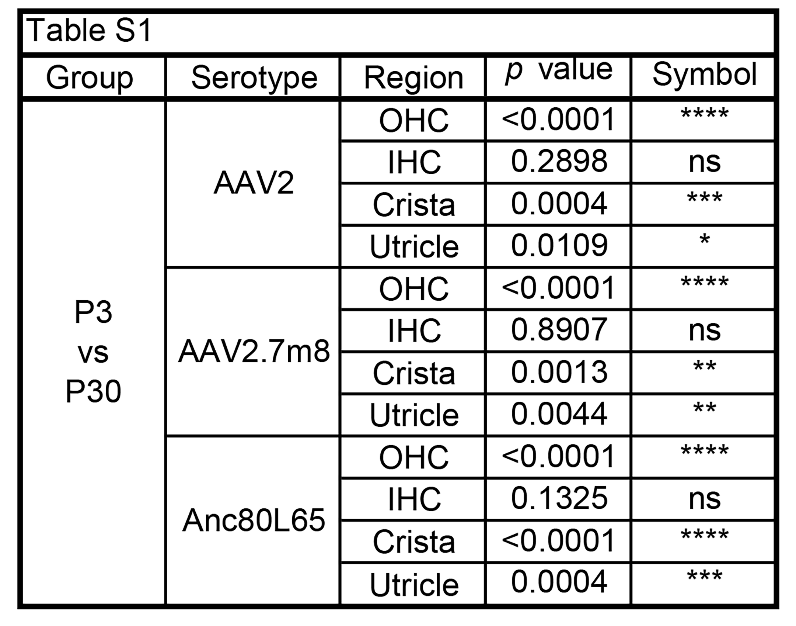


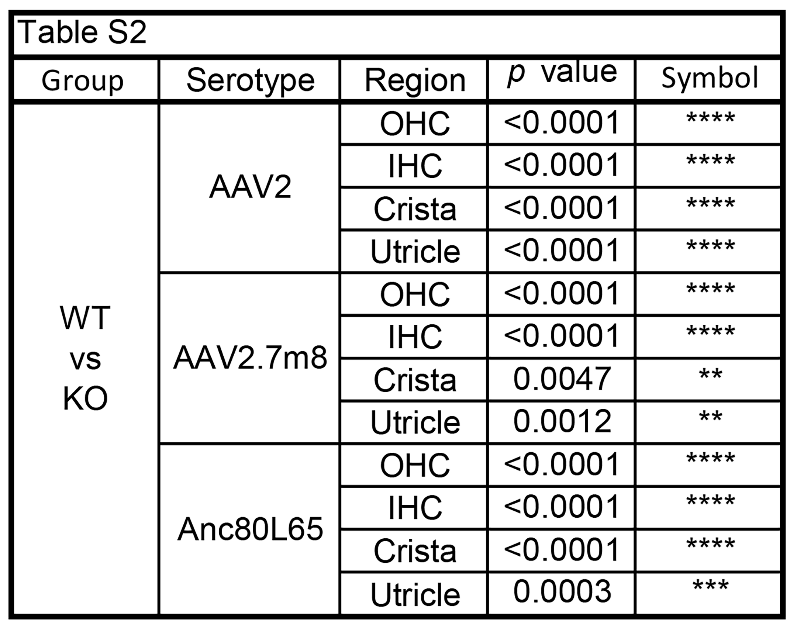


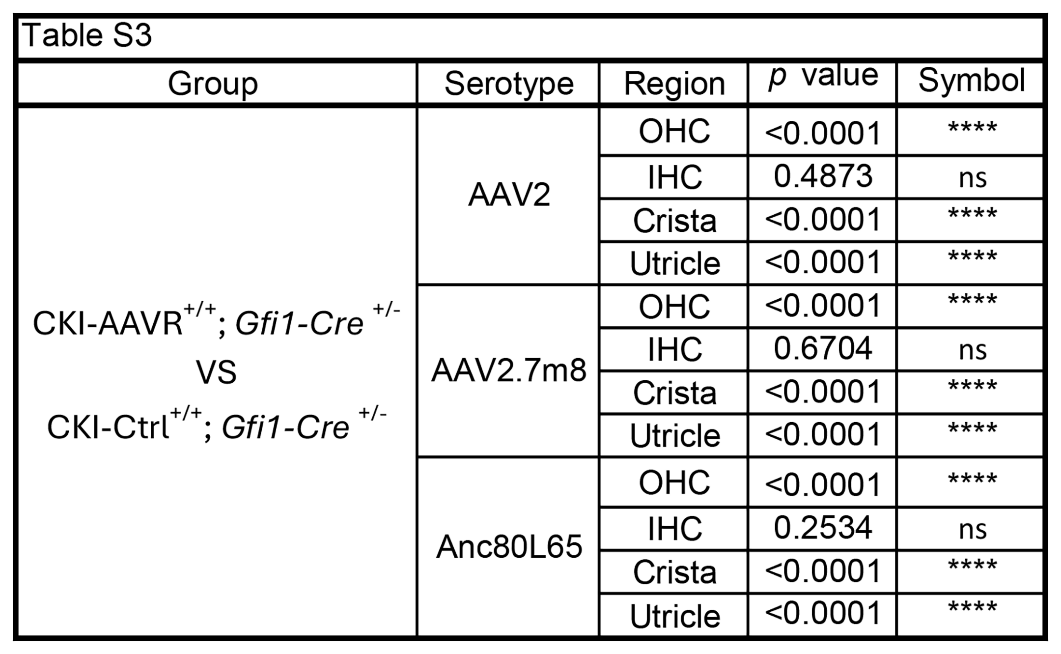


**References**

Chessum L, Matern MS, Kelly MC, Johnson SL, Ogawa Y, Milon B, et al. Helios is a key transcriptional regulator of outer hair cell maturation. Nature. 2018;563(7733):696-700, PMC6542691.

Li Y, Liu H, Giffen KP, Chen L, Beisel KW, He DZZ. Transcriptomes of cochlear inner and outer hair cells from adult mice. Sci Data. 2018;5:180199, PMC6167952.
